# Resveratrol alleviates lipopolysaccharide-induced inflammation in PC-12 cells and in rat model

**DOI:** 10.1186/s12896-019-0502-1

**Published:** 2019-01-28

**Authors:** Guiqi Zhang, Yi Liu, Lichen Xu, Chunhe Sha, Haibin Zhang, Weibing Xu

**Affiliations:** Department of Spinal Surgery, Dalian Municipal Central Hospital, No.826, Xinan Road, Shahekou District, Dalian, 116033 Liaoning China

**Keywords:** Inflammation, Resveratrol, miR-132, NF-κB, p38MAPK

## Abstract

**Background:**

Spinal cord injury (SCI) remains a huge medical problem nowadays as there is no hospital providing the versatile strategies for repairing central nervous system and restoring function. Herein, we focused on PC-12 cells as an important research tool and studied the potential role of resveratrol (RSV) in inflammation induced by LPS.

**Results:**

RSV improved inflammatory injury and functional recovery in rat model of SCI. RSV inhibited LPS-induced inflammatory injury in PC-12 cells via increasing viability, decreasing apoptosis, and suppressing IL-1β, IL-6, and TNF-α expression. miR-132 was down-regulated after LPS treatment but up-regulated after RSV administration. miR-132 silence curbed the protective effect of RSV. The results including increase of cell growth, suppression of inflammatory response, and blocking of NF-κB and p38MAPK pathways produced by RSV were all reversed by miR-132 silence.

**Conclusion:**

RSV could up-regulate miR-132 and further ameliorate inflammatory response in PC-12 cells by inhibiting NF-κB and p38MAPK pathways.

## Background

Spinal cord injury (SCI) has always been one of major medical problems due to it is really difficult to repair the central nervous system and recovery function [1]. Approximately half of SCI is induced by motor vehicle crashes and the other majorly results from falls, violence, and sports accident. SCI results in motor dysfunction, permanent paralysis and loss of sensation not only leading to serious long-term physical and psychological injuries but also causing serious social economic load [2]. The oxidative stress damage, posttraumatic inflammation, and motoneuron apoptosis and necrosis can be induced by SCI [3]. It is reported that if the SCI-induced inflammation is not suppressed, the neuronal dysfunction and cell death can be exaggerated [4, 5]. Thus, to modulate the inflammatory response is important for the treatment of SCI.

Many plant extracts have been used for medical treatment of inflammation. Resveratrol (RSV) is a natural product derived from peanuts, grapes, and many plants. The massive studies demonstrated the multiple functions and diverse activities of RSV, such as ameliorating aging-related metabolic phenotypes [6], improving insulin sensitivity and reducing oxidative stress in diabetic [7], and potently inhibiting cancer by mediating cell growth, apoptosis, angiogenesis, inflammation, and migration and metastasis by regulating multiple molecular targets [8]. More importantly, the role of RSV in SCI was investigated by a few studies, which indicated the characteristics of anti-inflammation and anti-apoptosis of RSV after SCI [9–11].

microRNA (miRNA), the small noncoding RNA, is regarded as a new paradigm for understanding inflammation diseases because miRNAs are important regulators in many biological processes [12, 13]. microRNA-132 (miR-132) has been shown to participate in the regulation of inflammation [14, 15]. One study explained that miR-132 suppressed lipopolysaccharide (LPS)-induced inflammation in alveolar macrophages via enhancing the cholinergic anti-inflammation pathway [14]. miR-132 was related to Toll-like receptor 4 (TLR4), regulated astrocyte activation, and modulated innate immune response in neurological diseases [16]. However, the protective effect of miR-132 on PC-12 cells has been rarely reported. The regulatory role of miR-132 in the effect of RSV was examined in this study.

Herein, we assessed the anti-inflammatory effect of RSV on the rat model of SCI or on LPS-treated PC-12 cells. The molecule mechanism of RSV in repression of inflammation response was also investigated. How miR-132 regulated the function of RSV in PC-12 cells was explored.

## Results

### RSV improved inflammatory injury and functional recovery in rat model of SCI

**Fig. 1:**
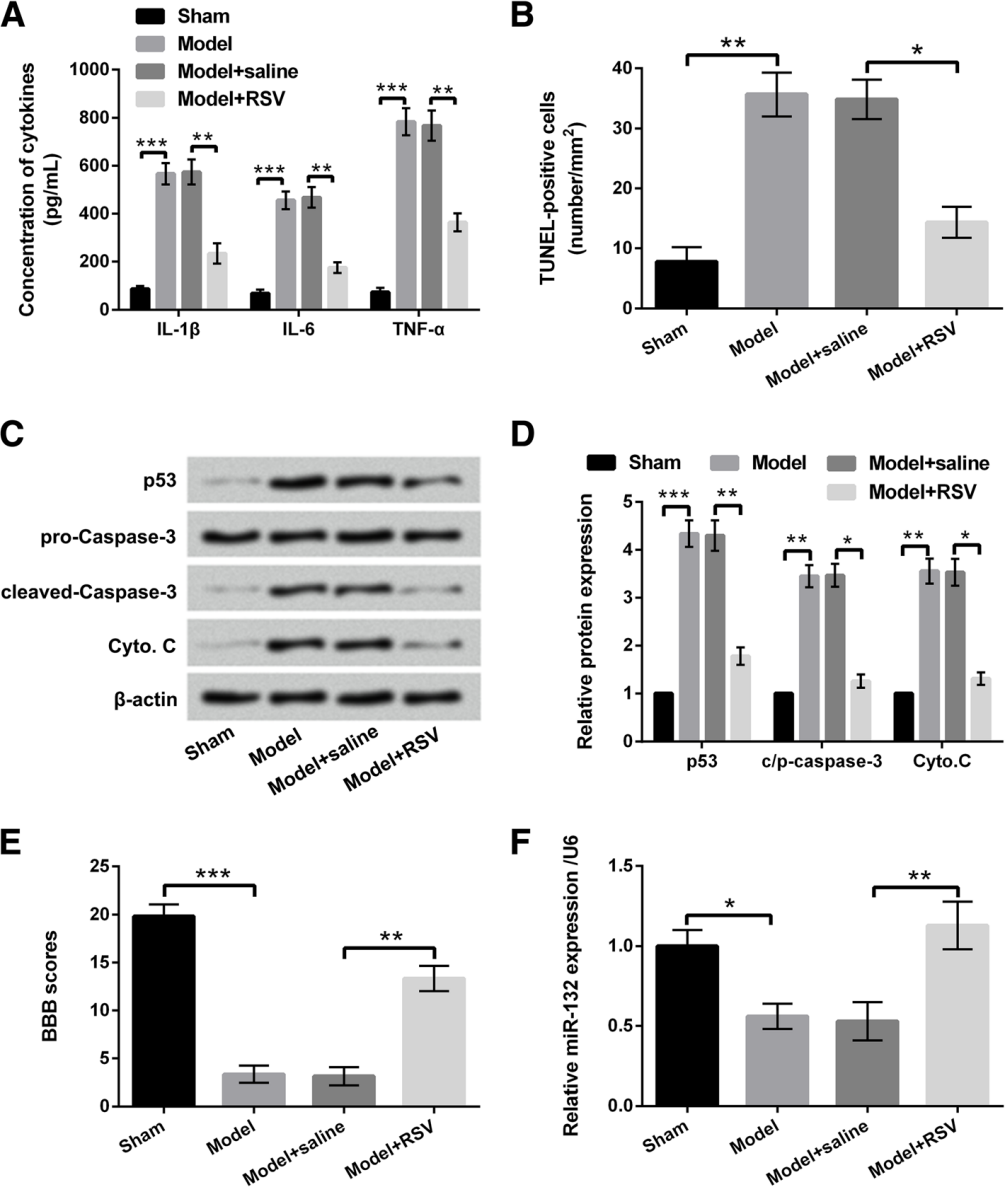
RSV improved inflammatory injury and functional recovery in rat model of SCI. A total of 40 SD rats were randomly divided into four groups: Sham group, Model group, Model+saline group and Model+RSV group. **a** The concentrations of IL-1β, IL-6, and TNF-α were increased in Model group, but decreased in Model+RSV group. **b** The number of TUNEL-positive cells and (**c** and **d**) the pro-apoptotic protein expressions were all enhanced in Model group, but declined in Model+RSV group. **e** The BBB scores were decreased in Model group, but increased in Model+RSV group. **f** The expression level of miR-132 was down-regulated in Model group, but up-regulated in Model+RSV group. * *P* < 0.05, ** *P* < 0.01, *** *P* < 0.001

Animal experiments were carried out to explore whether RSV exerted the protective effect on SCI. The rats were divided into four groups, including Sham group, Model group, Model+saline group and Model+RSV group. Results in Fig. 1a showed that the concentrations of IL-1β, IL-6 and TNF-α were significantly increased in SCI Model group as relative to Sham group (*P* < 0.001). Moreover, cell apoptosis analytical results displayed that TUNEL-positive cells and expression of pro-apoptotic regulators, p53, cleaved-Caspase-3 and Cyto. C were all promoted in Model group (*P* < 0.01 or *P* < 0.001, Fig. 1b-d). However, in Model+RSV group, we observed that the concentrations of pro-inflammatory cytokines, TUNEL-positive cells and apoptosis-associated factors were all inhibited, as compared to Model+saline group (*P* < 0.05 or *P* < 0.01, Fig. 1b-d). Additionally, evaluation of neural function showed that BBB scores in Model group were significantly declined compared to Sham group (*P* < 0.001). However, BBB scores in Model+RSV group were significantly increwased compared to Model+saline group (*P* < 0.01, Fig. 1e). More interestingly, qRT-PCR analytical results demonstrated that the expression level of miR-132 was down-regulated in Model group compared with Sham group (*P* < 0.05), but up-regulated in Model+RSV group compared to Model+saline group (*P* < 0.01, Fig. 1f). These data indicated that RSV could improve neuron protection and functional recovery in rat model of SCI. Moreover, miR-132 might be a key regulator in this process. More in vitro experiments were performed to further investigate the effect of RSV on SCI.

### RSV suppressed LPS-induced PC-12 cell injury

**Fig. 2:**
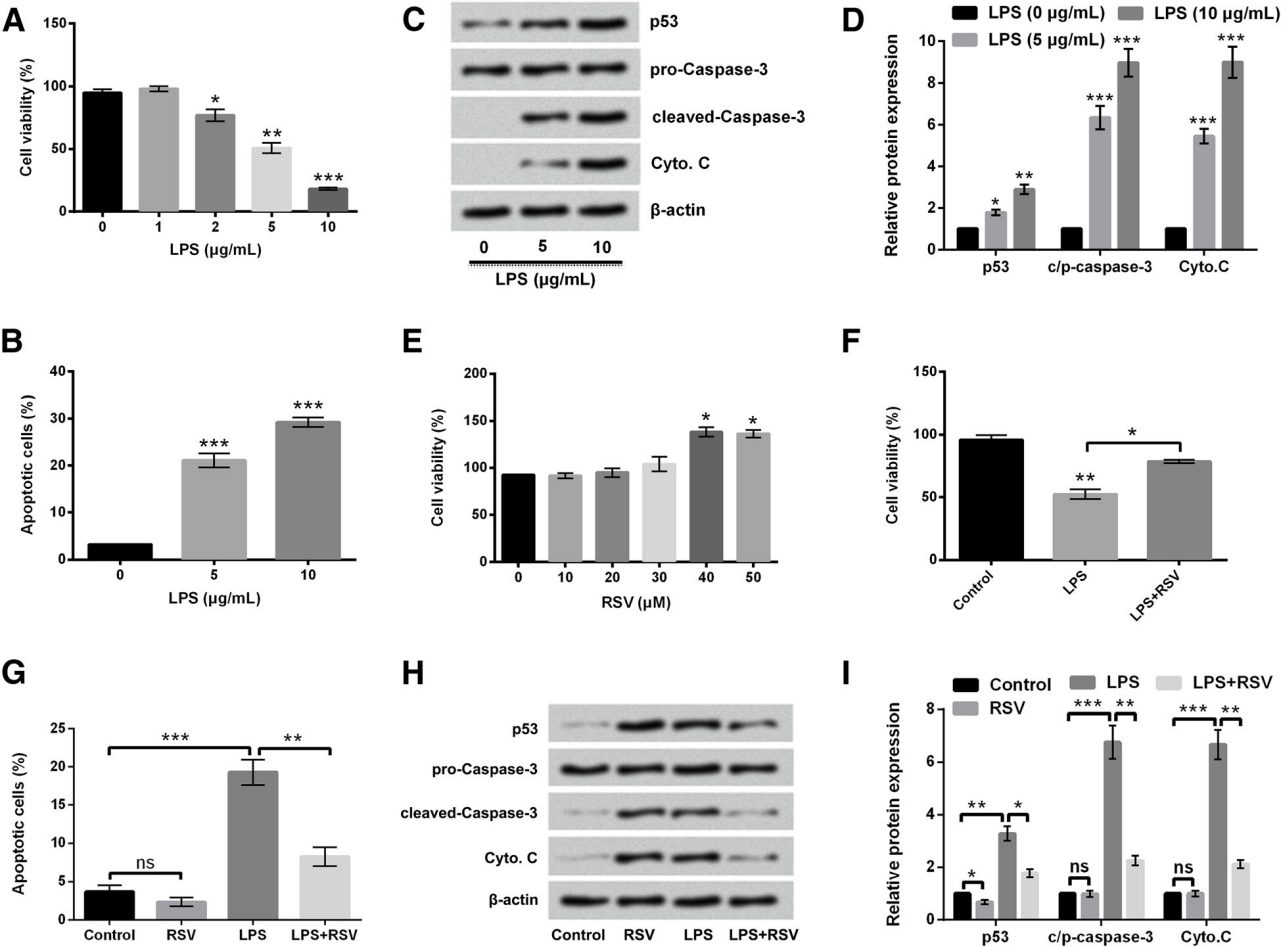
RSV inhibited LPS-induced loss of viability and increase of apoptosis. LPS **a** decreased viability, **b** increased apoptotic cell rate, and (**c** and **d**) enhanced pro-apoptotic protein levels. **e** RSV promoted cell viability when applied higher than concentration of 40 μM. RSV with dose of 30 μM **f** improved cell viability, **g** reduced apoptotic cell rate, and (**h** and **i**) diminished pro-apoptotic protein levels. * *P* < 0.05, ** *P* < 0.01, *** *P* < 0.001

Cell viability was decreased by LPS treatment in a dose dependent manner (Fig. 2a). Changes of cell apoptosis were also investigated by detecting apoptotic cell rate and apoptotic factor expressions. Results showed that LPS treatment increased apoptotic cell rate (Fig. 2b) and expression levels of pro-apoptotic protein (Fig. 2c and d). Next, the effects of RSV with different concentrations on PC-12 cell viability were analyzed. Due to the viability-promoting effect of high dose of RSV (Fig. 2e), concentration of 30 μM was used for subsequent studies. Then, we analyzed the effects of RSV on LPS-treated PC-12 cells. Data demonstrated that RSV attenuated the effects of LPS on cell viability and apoptosis. Cell viability was improved in response to RSV (*P* < 0.05, Fig. 2f). Meanwhile, apoptotic cell rate was decreased (*P* < 0.01, Fig. 2g) and the expression levels of pro-apoptotic regulators, p53, cleaved-Caspase-3, and Cyto. C were all inhibited in LPS and RSV co-treated cells (Fig. 2h and i). There was no obvious change of cell apoptosis and apoptosis-associated factor in RSV alone treated cells. (Fig. 2g-i). These data suggest that RSV repressed LPS-induced loss of viability and increase of apoptosis.

### RSV administration inhibited LPS-induced up-regulations of pro-inflammatory factors

**Fig. 3:**
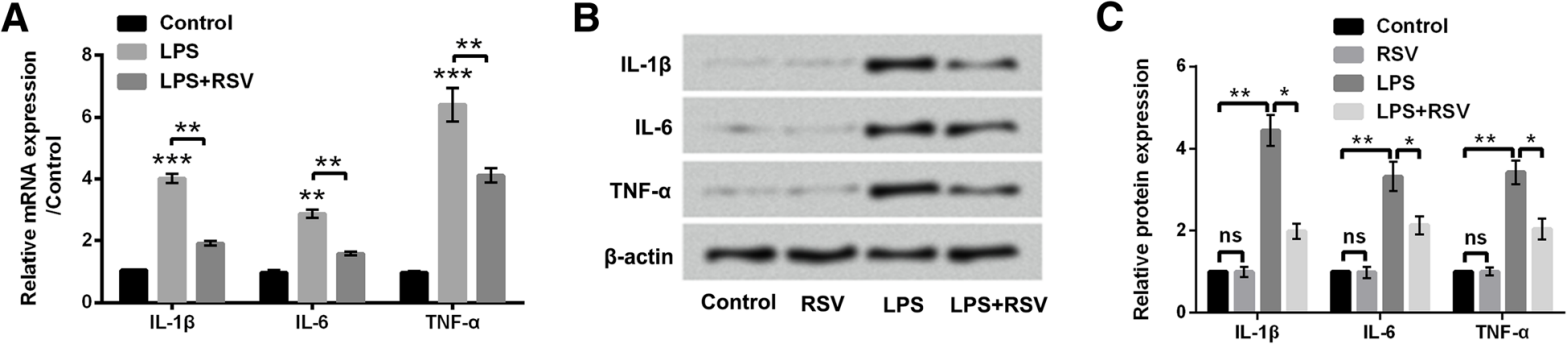
RSV inhibited LPS-induced overexpression of pro-inflammatory cytokines. LPS treatment induced (**a**) mRNA and (**b** and **c**) protein levels of IL-1β, IL-6, and TNF-α. RSV suppressed (**a**) mRNA and (**b** and **c**) protein levels of IL-1β, IL-6, and TNF-α. * *P* < 0.05, ** *P* < 0.01, *** *P* < 0.001, ns, no significant difference

The inflammatory response in PC-12 cells was evaluated by detecting expressions of pro-inflammatory factors (IL-1β, IL-6, and TNF-α) after LPS or/and RSV treatment. IL-1β, IL-6, and TNF-α were all up-regulated in mRNA expression levels after LPS treatment (*P* < 0.01 or *P* < 0.001, Fig. 3a). However, all their expression levels were suppressed in RSV treated cells (*P* < 0.01, Fig. 3a). Similarly, the protein levels of IL-1β, IL-6, and TNF-α were all increased in LPS-treated cells, but decreased in RSV and LPS co-treated cells (*P* < 0.05 or *P* < 0.01, Fig. 3b and c). These data implied that RSV repressed LPS-induced inflammatory injury in PC-12 cells.

### miR-132 modulated the effects of RSV on PC-12 cells

**Fig. 4:**
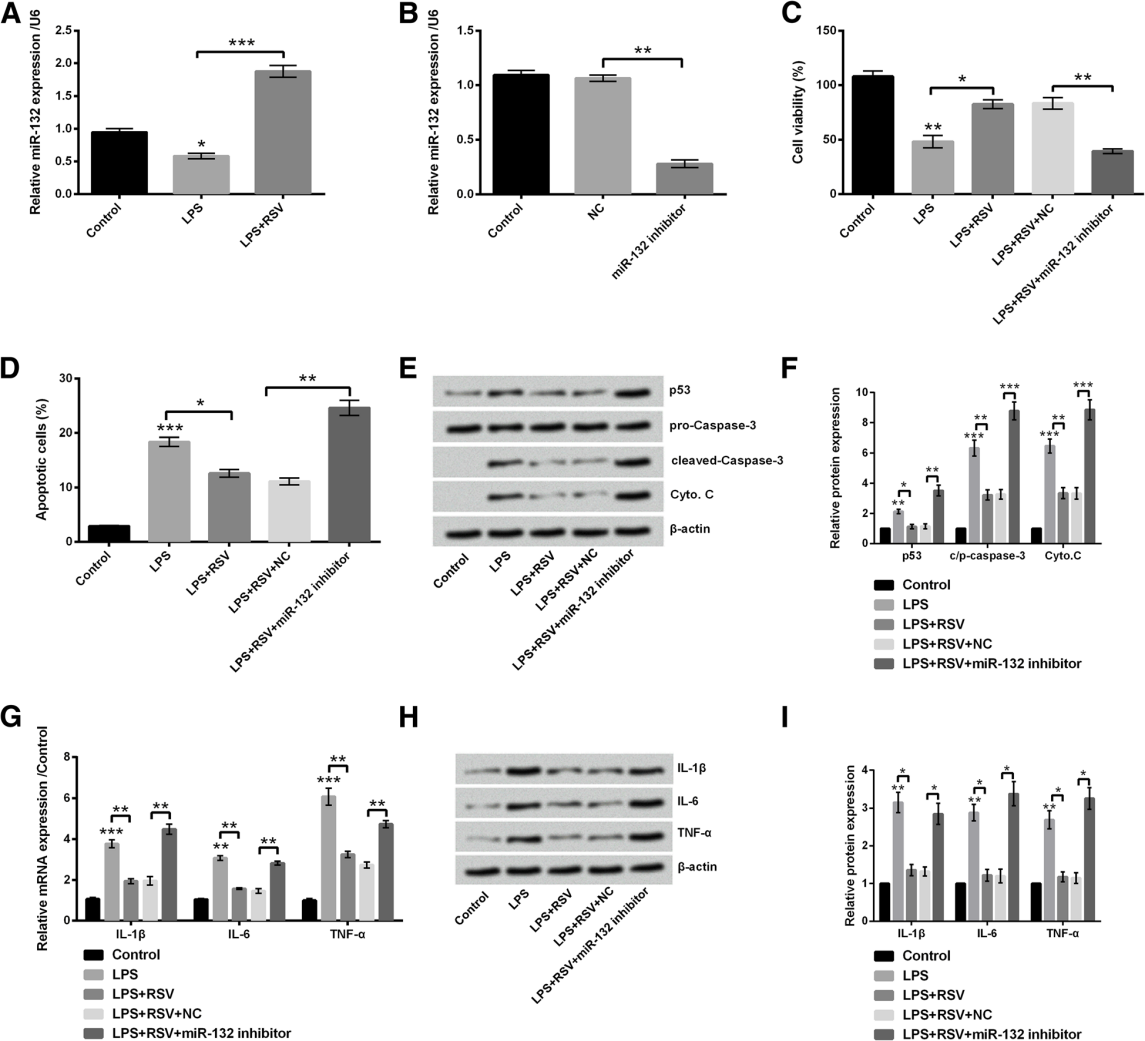
RSV ameliorated LPS injury by up-regulating miR-132. **a** miR-132 expression was down-regulated after LPS treatment but was increased after RSV administration. **b** miR-132 expression was decreased after determining miR-132-inhibitor-transfection efficiency. miR-132 knockdown **c** inhibited RSV-induced increase of viability, **d** decrease of apoptotic cell rate, as well as (**e** and **f**) suppression of pro-apoptotic protein level, and (**g**) mRNA/(H and I) protein levels of IL-1β, IL-6, and TNF-α. * *P* < 0.05, ** *P* < 0.01, *** *P* < 0.001

For clarifying the anti-inflammatory mechanism of RSV, the role of miR-132 was analyzed. miR-132 expression was significantly declined in LPS-treated cells (*P* < 0.05), whereas, its expression was elevated in response to RSV treatment (*P* < 0.001, Fig. 4a). The modulating effects of miR-132 on growth and inflammatory reaction in PC-12 cells were detected as its expression was altered by LPS and RSV. Before this, cells were transfected with miR-132 inhibitor, and miR-132 expression was decreased after transfection (*P* < 0.01, Fig. 4b). According to our data, miR-132 silence impaired the protective effects of RSV on PC-12 cells. In concrete terms, significant reduction of cell viability (*P* < 0.01, Fig. 4c) and increase of apoptotic cell rate (*P* < 0.01, Fig. 4d), augmented the expression levels of pro-apoptotic regulators (*P* < 0.05, P < 0.01 or *P* < 0.001, Fig. 4e and f), as well as high levels of IL-1β, IL-6, and TNF-α (*P* < 0.05 or *P* < 0.01, Fig. 4g-i) were found in miR-132 silence group. Thus we speculated that RSV might ameliorate LPS-induced cell injury by up-regulating miR-132.

### RSV inhibited NF-κB and p38MAPK signaling pathways by up-regulating miR-132

**Fig. 5:**
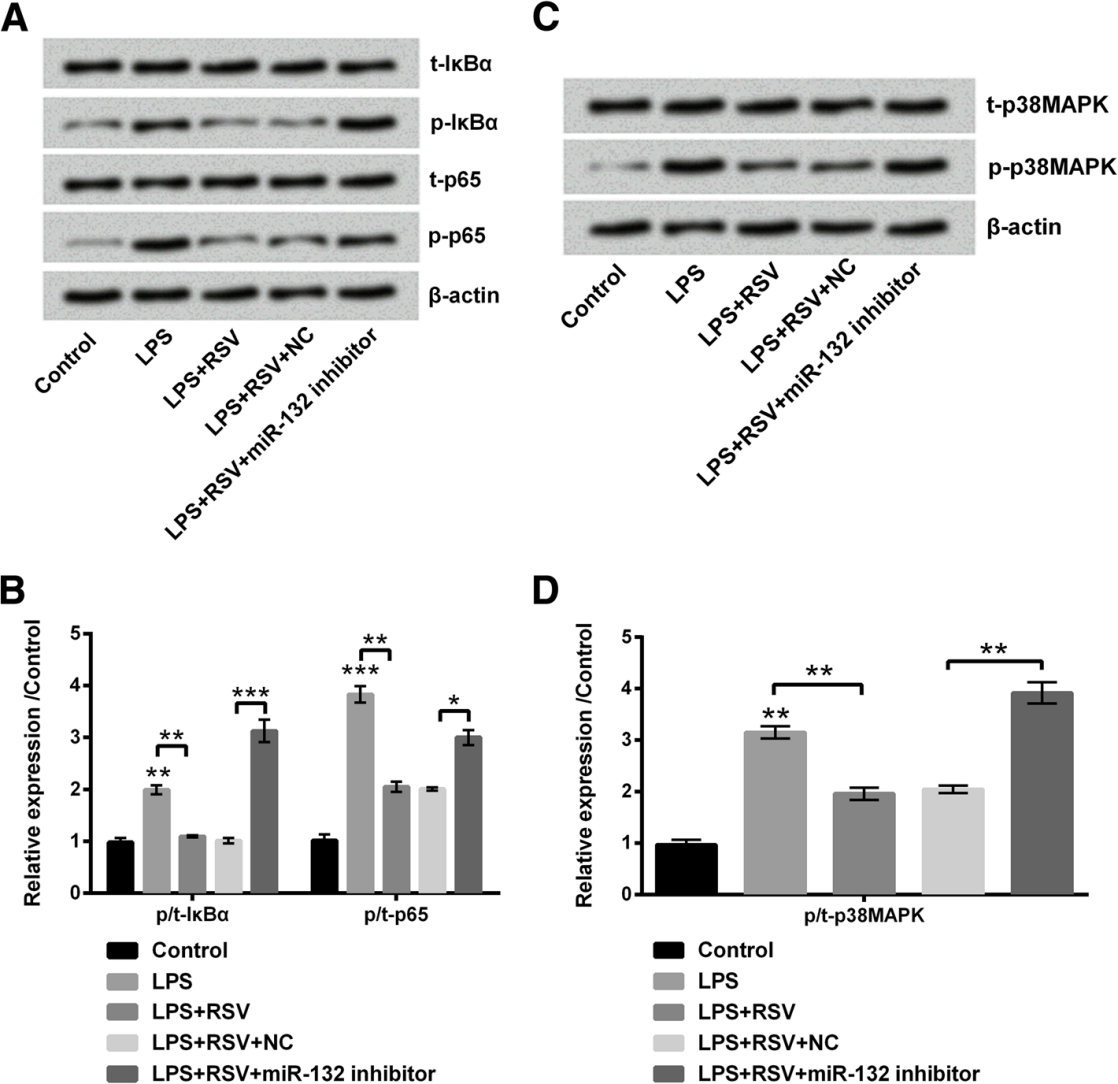
RSV inhibited NF-κB and p38MAPK pathways. (**a** and **b**) NF-κB signaling pathway was inhibited after RSV administration but activated after miR-132 silence. (**c** and **d**) p38MAPK signaling pathway was inhibited after RSV administration but activated after miR-132 silence. * *P* < 0.05, ** *P* < 0.01, *** *P* < 0.001

Whether some signaling pathways participated in the protective function of RSV was not clear. Thus we further explored this in the current study. The activations of NF-κB pathway and p38MAPK pathway were analyzed by Western blot (Fig. 5a-d). It is well-known that NF-κB and p38MAPK pathways can be activated by LPS. We next found that NF-κB nd p38MAPK pathways were suppressed by RSV because of the decreased the protein levels of p-IκBα, p-p65, and p-p38MAPK (all *P* < 0.01). However, the protein levels of p-IκBα, p-p65, and p-p38MAPK were all enhanced after miR-132 was knocked down (*P* < 0.001, *P* < 0.01, and *P* < 0.05), suggesting that NF-κB and p38MAPK pathways might be suppressed by RSV via up-regulation of miR-132.

## Discussion

Abundant studies focus on the therapies of SCI as SCI is a high-cost, low-curative ratio neurological disability. Inflammatory reaction incited by SCI contributes substantially to the secondary effect, with both beneficial effect and devastating effect [17]. Inhibiting harmful inflammatory response possibly contributes to protect from aggravation of the secondary injury cascade and further deterioration of neurological function [18]. Meanwhile, reducing neuronal and glial apoptosis with concomitant promotion of neurological function recovery is also associated with therapeutic targets in SCI [19]. Therefore, the aim of application of RSV in this study was to limit the inflammatory response and inhibit cell apoptosis following SCI.

Firstly, animal experiments were carried out to explore the effect of RSV on the rat model of SCI. Interesting results showed that RSV improved inflammatory injury and functional recovery in rat model of SCI. Additionally, in vitro experiment revealed that the cell injury was evaluated after LPS treatment. Cell viability and apoptosis were respectively suppressed and potentiated. The well-known apoptosis-related genes were found to be up-regulated, including p53, cleaved-Caspase-3, and Cyto. C. Moreover, the expression levels of pro-inflammatory factors were increased. However, RSV was found to reverse the negative effects mentioned above in PC-12 cells. RSV treatment produced viability-promoting and apoptosis-inhibitory effects and suppressed expressions of tested pro-inflammatory cytokines. Many investigations show that RSV possesses various biological activities, including anti-inflammatory effect [20]. RSV was showed to reduce growth inhibition, restrain apoptosis, and suppress isoflurane-induced inflammation and oxidative stress in PC-12 cells [21]. In one previous study, RSV was used to treat experimental SCI and data showed that decreased TNF-α and IL-1β expression levels, reduced TUNEL positive cells, and increased glial and motor neuron cells were found in RSV-treated group [22]. RSV potentiated the functional recovery of rat dorsal neuronal following SCI due to its activities of anti-oxidation, anti-inflammation and anti-apoptosis [9]. Therefore, all of these data were consistent with our study.

RSV displayed an important role in improving viability, reducing apoptosis, and suppressing inflammation, but how it exerted these effects was not clearly described. RSV could increase mitochondrial biogenesis and function and also protect against metabolic decline [23]. A series of miRNAs have been reported to be involved in different functions of RSV, like miR-138 [24], miR-17 [25], and miR-221 [26]. This study showed that miR-132 expression was down-regulated in LPS-stimulated cells but up-regulated after RSV treatment. miR-132 suppression impaired the effects of RSV on PC-12 cells, which indicated that the protective effect of RSV might be related to miR-132 up-regulation. Similarly, miR-132 was showed to attenuate inflammatory injury incited by LPS via targeting TRAF6 in neuron HT-22 cells [27]. miR-132 is a critical regulator mediating the integration of newborn neurons into the adult dentate gyrus. Besides, miR-132 silence in PC-12 cells induced elevated the expression levels of hundreds of genes, mostly inflammatory/immune signaling-associated genes [28]. These studies were not in conflict with our study.

Further mechanism research demonstrated that LPS-induced activations of NF-κB and p38MAPK pathways were inhibited by RSV and miR-132 up-regulation might contribute to this inhibitory effect of RSV. It is well-known that NF-κB signal pathway is an inducer of LPS as well as p38MAPK signal [29–31]. miR-132 attenuated LPS-induced inflammation via inhibiting the NF-κB and MEK/ERK pathways [27]. RSV suppressed the NF-κB inflammation pathway in prevention of fatty liver disease [32] and attenuated neuronal autophagy and inflammatory reaction by inhibiting the NF-κB signal in experimental traumatic brain injury [33]. RSV could also suppress the MAPK signal pathway in LPS-induced mice mastitis [34]. NF-κB p65 and p38 MAPK were key downstream factors of TLR4 and RSV was showed to block expression of TLR4 and suppress the phosphorylation of NF-κB p65 and p38 MAPK [35]. These data were consistent with our study.

## Conclusions

Taken together, these findings demonstrated that RSV modulated miR-132 expression and attenuated LPS-induced inflammation in PC-12 cells and in rat model via promoting cell growth and suppressing expression levels of several pro-inflammatory cytokines. RSV exerted the anti-inflammation property by blocking NF-κB and p38 MAPK signaling pathways, which were also mediated by miR-132. RSV might reduce secondary damage after SCI.

## Materials and methods

### Animal and experimental groups

A total of 40 SD rats (280–320 g) were obtained from Shanghai Laboratory Animal Center of Chinese Academy of Sciences (Shanghai, China). These rats were raised in cages with water ad libitum. Experimental protocols were approved by the Ethics Committee of Dalian Municipal Central Hospital. Above rats were randomly divided into four groups: Sham group, Model group, Model+saline group and Model+RSV group. The model of SCI was established according to the model of Allen’s weight hit and the study from Liu et al [36, 37]. These rats were given intraperitoneal injection of trichloroacetaldehyde (300 mg/kg, i.p., Sigma-Aldrich, St. Louis, USA). Subsequently, the anesthetized rats were laid flat on the operating table, and the furs were shaved around chest and abdomen. The experiment area of rats was disinfected with alcohol, and a 3 cm incision was made at the center of the eighth thoracic spine to expose the spinal dura mater. The hitting area with the size of 3 mm × 4 mm exposed at the center of the spinal cord. After hitting, the striking instrument was quickly removed and the wound was sealed with suture. Rats in the Model+saline group received spinal cord injury and saline injection. Rats in the Model+RSV group received RSV treatment (200 mg/kg, i.p., Sigma-Aldrich) three times per day for 3 days after the injury. Rats in the Sham group received same surgical procedures but no trauma hit and RSV treatment. Finally, these rats were sacrificed by intraperitoneal injection of 100–150 mg/kg sodium pentobarbital (Sigma-Aldrich) 3 days after surgery. We tried our best to alleviate the pain of these rats.

### Enzyme-linked immunosorbent (ELISA) assay

The concentrations of interleukin-1 beta (IL-1β), interleukin-6 (IL-6) and tumor necrosis factor-α (TNF-α) in Sham group, Model group, Model+saline group and Model+RSV group were examined by using ELISA assay based on the manufacturer instruction. In brief, the serum from the four experimental groups was collected, and the concentrations of IL-1β, IL-6 and TNF-α were measured by the corresponding ELISA kits (R&D Systems, Abingdon, UK).

### TUNEL staining

TUNEL Apoptosis Detection Kit (Promega, Madison, WI, USA) was performed to detect apoptotic neurons. The number of TUNEL-positive cells was counted in five randomly chosen fields within each slide at 400× with an optic microscope (Olympus, Tokyo, Japan). The index of apoptosis was calculated according to the ratio of overall apoptotic cells and the total cells.

### Evaluation of neuronal function recovery

Basso-Beattie-Bresnahan (BBB) score method was used to evaluate the functional recovery of rats in Sham group, Model group, Model+saline group and Model+RSV group. The scoring range of BBB is from zero (complete paralysis) to twenty one (normal locomotion). After 72 h post-surgery, the locomotion activity of hindlimb was evaluated.

### Cell culture and treatment

PC-12 cells were purchased from the American Type Culture Collection (ATCC, Rockville, MD, USA). They were cultured in DMEM (1 × 10^4^ cells/ml) supplemented with fetal bovine serum (10% (*v*/v)), penicillin (100 U/mL) and streptomycin (100 μg/mL) at 37 °C in the incubator under suitable humidity condition. Cells were treated with LPS (1, 2, 5, and 10 μg/mL) for 12 h and RSV (ref: R5010, ≥99%, Sigma-Aldrich, St. Louis, USA) at concentrations ranging from 10 μM to 50 μM.

### CCK-8 assay

CCK-8 assay was conducted to determine cell viability. Approximately 5 × 10^3^ cells were seeded in each well of 96-well plate. After treatments, 10 μL of CCK-8 solution (Beyotime, Shanghai, China) was added to the culture medium. After further incubation for 2 h, the absorbance was measured at 450 nm using a Microplate Reader (Bio-Rad, Hercules, CA, USA).

### Apoptosis assay

PC-12 cells were gathered by centrifugation (2000 *g* for 10 min) and then they were rinsed with PBS. Afterwards, PC-12 cells were suspended in 300 μL binding buffer followed by adding 5 μL Annexin V-FITC solution. Then cells were incubated with Annexin V-FITC at room temperature in the dark for 15 min. After incubation, 5 μL PI solution was added in the cell suspension. Finally, 200 μL binding buffer was supplemented and flow cytometry analysis was conducted immediately. FACScan (Beckman Coulter, Fullerton, CA, USA) was used and the data were analyzed by using FlowJo software.

### miRNA transfection

For miR-132 silence transfection, miR-132 inhibitor (5’-AGU AAC AAU CGA AAG CCA CGG U-3′) synthesized by GenePharma Co (Shanghai, China) was transfected into cells using Lipofectamine 3000 reagent (Invitrogen, Carlsbad, CA, USA) in this study. Transfected cells were harvested after post-transfection of 48 h.

### qRT-PCR analysis

The qRT-PCR for miR-132 was performed in a CFX96 Real-Time PCR Detection System (Bio-Rad) as described previously [38]. The primer sequences for miR-132 were followed as: forward, 5’-ACC GTG GCT TTC GAT TGT TA-3′; reverse, 5’-GGC GAC CAT GGC TGT AGA CT-3′. Total miRNAs in treated or transfected cells was extracted by using miRNeasy Mini Kit (Qiagen, Shenzhen, China). RNAs were reverse transcribed with PrimeScript Reverse Transcriptase (Takara, Dalian, China). For mRNA levels of IL-1β, IL-6, and TNF-α, total RNAs were extracted by using Trizol reagent and treated with DNAse I (Promega, Madison, WI, USA), and were reverse transcribed in a reaction system containing random primers and M-MLV reverse transcriptase. Subsequently, the cDNAs were amplified by using RT-PCR with SYBR green Master Mix. U6 was used as the internal control for miR-132 expression analysis and β-actin was used as the internal control for determining expression of IL-1β, IL-6, and TNF-α. Their expression levels were calculated by relative quantification 2^−△△CT^ method.

### Western blot

Total protein was extracted from PC-12 cells; and after quantification, 50 μg protein was separated by 12% SDS-PAGE and transferred to the PVDF membrane. The membranes were incubated with appropriate primary antibodies prepared in 5% blocking buffer at a dilution of 1:1000 at 4 °C overnight. The primary antibodies used in this study were displayed as follows: p53 (ab26), pro-Caspase-3 (ab44976), cleaved-Casapse-3 (ab13847), Cyto. C (ab133504), IL-1β (ab9722), IL-6 (ab9324), TNF-α (ab6671), IκBα (ab32518), p-IκBα (S36, ab133462), p65 (ab16502), p-p65 (S536, ab86299), p38MAPK (ab31828), p-p38MAPK (T180, ab178867), and β-actin (ab8226, Abcam, Cambridge, MA, USA). After incubation, the PVDF membranes were further incubated with the horseradish peroxidase-conjugated second antibody for 1 h at room temperature. Subsequently, signals were captured by using the enhanced chemiluminescence reagent of Lumi-Light Western Blotting Substrate (Sigma-Aldrich, St, Louis, MO, USA), and were scanned by using the UMAX Vista S6E Flatbed Scanner (UMAX data systems inc., Hsinchu, Taiwan). The intensity of the target band was analyzed by Image Lab™ Software (Bio-Rad, Hercules, CA, USA).

### Statistical analysis

Data were expressed as the mean ± SD of at least three independent experiments. We evaluated the data with a one-way analysis of variance (ANOVA) followed by Sidak’s post hoc test for multiple comparisons. The data with *P* value less than 0.05 was considered significant.

## Abbreviations

LPS: Lipopolysaccharide
miR-132: microRNA-132
miRNA: microRNA
RSV: Resveratrol
SCI: Spinal cord injury
